# Steroid-Resistant Rash With Neuropsychiatric Deterioration and Weight Loss: A Modern-Day Case of Pellagra

**DOI:** 10.7759/cureus.92437

**Published:** 2025-09-16

**Authors:** Harshita Ryali, Hagar Elgezeri, Humaira Ahmed, Daniel Keith, Charankumal Thandi

**Affiliations:** 1 Dermatology, North Bristol NHS Trust, Bristol, GBR; 2 Faculty of Medicine, Cairo University, Cairo, EGY; 3 Dermatology, Gloucestershire Hospitals NHS Foundation Trust, Gloucester, GBR; 4 Dermatology, University of Bristol, Bristol, GBR

**Keywords:** acute delirium, casal’s necklace, gastrointestinal, generalised weakness, medical dermatology, nutritional deficiency, photosensitive rash, vitamin b3 deficiency

## Abstract

Pellagra is a multisystem disorder caused by niacin (vitamin B3) deficiency, which typically presents with dermatitis, diarrhoea, and dementia. Although it has become less prevalent in developed countries due to improved nutrition and the fortification of foods with niacin, isolated cases continue to emerge among vulnerable groups. We present a case of a 50-year-old woman with a complex psychiatric and neurological history who developed a widespread, scaly, erythematous rash unresponsive to corticosteroids, along with progressive dysphagia, diarrhoea, and neurocognitive decline. Initial investigations and management focused on suspected dermatomyositis. However, the clinical picture evolved despite steroid treatment and extensive investigations. Following the discovery that she followed a restrictive ketogenic diet over several years, a clinical diagnosis of pellagra was made, and treatment with nicotinamide and B-vitamin supplementation led to rapid and sustained resolution of all her symptoms. This case underscores the importance of considering pellagra in patients presenting with neuropsychiatric and dermatologic symptoms, even in modern healthcare settings. Early recognition is essential as untreated pellagra can be fatal, despite the fact that treatment with nutritional therapy is straightforward.

## Introduction

Pellagra is a multisystem disorder caused by a deficiency of niacin (vitamin B3). Although it has become less prevalent in developed countries due to improved nutrition and the fortification of foods with niacin, isolated cases continue to emerge among vulnerable groups. Niacin is a part of nicotinamide adenine dinucleotide (NAD) and nicotinamide adenine dinucleotide phosphate (NADP) and is crucial for energy production, metabolic processes, DNA repair, and antioxidant function. Its deficiency particularly affects tissues with high energy requirements or cell turnover rate, such as the skin, bowel, and brain [1]. Pellagra is characterised by dermatitis, diarrhoea, and dementia, which, if left untreated, may lead to death.

This case report was previously presented as a poster at the European Academy of Dermatology and Venereology (EADV) Symposium 2025, held in Prague on 22-24 May 2025, titled "Confused patient with dysphagia, psychosis, neuropathy, weight loss, and a progressing widespread rash unresponsive to steroids - an uncommon case of pellagra."

## Case presentation

A 50-year-old woman presented with a few months’ history of a scaly, erythematous, and painful rash affecting her face, neck, torso, and limbs. She had a history of epilepsy with a previous partial temporal lobectomy, bipolar affective disorder, anxiety, depression, eating disorder, and (well-controlled) psoriasis. The rash initially appeared in a shawl-like, photosensitive distribution, suspicious for dermatomyositis. A skin biopsy was done, which highlighted a subtle interface dermatitis without significant dermal inflammation; this seemed to support dermatomyositis as a histological differential. She was prescribed topical mometasone ointment for the body, betamethasone cream for the face, and given 40 mg of oral prednisolone. Her rash, however, continued to spread, and she experienced progressive generalised weakness, unsteady gait, along with new diarrhoea and dysphagia, which necessitated her admission as an inpatient.

Her investigations revealed anaemia with haemoglobin of 104 g/L. Serum vitamin B12 was found to be borderline deficient at 247 ng/L, whereas folate, ferritin, vitamins D, A, and E, selenium, copper, and zinc were normal (Table 1). Hepatitis B and C, HIV, syphilis serology, and autoimmune screen were negative. Her CT head was normal, and CT thorax, abdomen, and pelvis did not show evidence of malignancy. Tumour marker and paraneoplastic screen were negative. Creatinine kinase was normal at 20 U/L, and the myositis panel was negative. MRI of the lower limb showed no muscle oedema to suggest myositis.

**Table 1:** Summary of patient's laboratory results during admission.

Test	Result	Reference range
Haemoglobin (g/L)	104	120 - 150
Vitamin B12 (ng/L)	247	180 - 914
Folate (µg/L)	6.1	3.0 - 20.0
Ferritin (µg/L)	16	11 - 307
Vitamin D (nmol/L)	93	>50
Vitamin A (µmol/L)	2.8	0.8 - 3.0
Vitamin E (µmol/L)	43.3	10.2 - 39.0
Selenium (µmol/L)	0.89	0.75 - 1.46
Copper (µmol/L)	20.6	11.0 - 25.0
Zinc (µmol/L)	7.8	10.0 - 20.0
Creatinine kinase (U/L)	20	25 - 200

**Figure 1:**
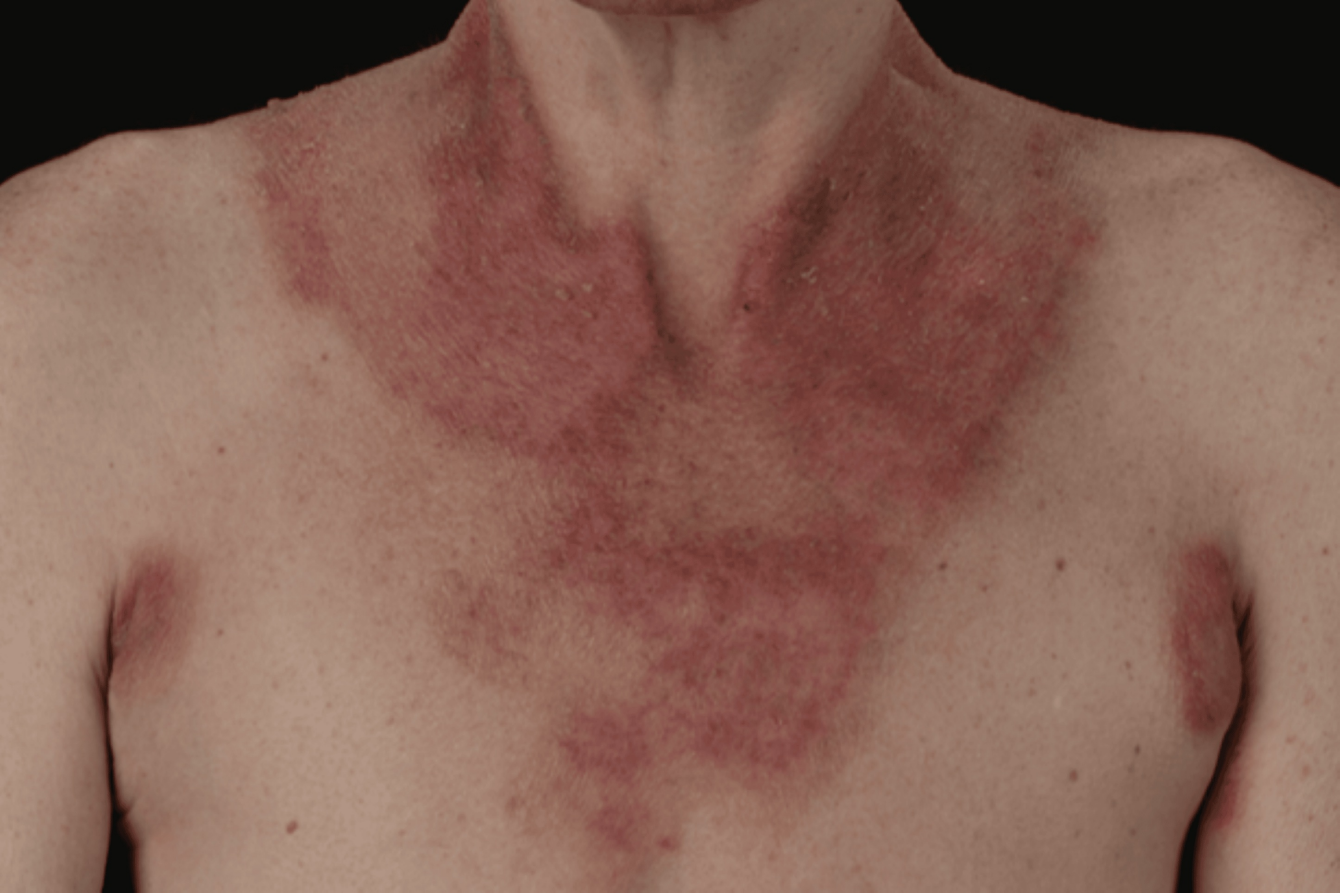
Anterior view of the band of dermatitis encircling the neck (Casal's necklace) This is a photosensitive dermatitis. The upper border of the dermatitis at the neck is spared as light exposure is obstructed by the chin, while the lower border is dependent on the clothing of the patient.

**Figure 2:**
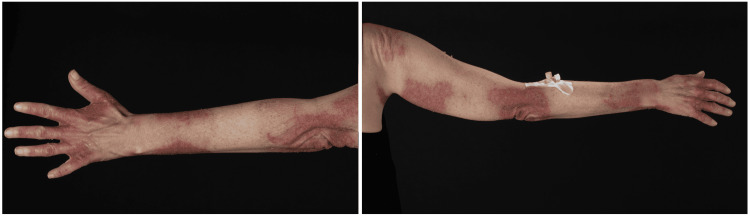
Dermatitis of the left (left panel) and right (right panel) upper limbs. The distribution of the rash is as a bilateral, symmetrical, and well-demarcated dermatitis. It may initially present similarly to sunburn, with redness and swelling, and later develop scaling and hyperpigmentation. This condition is sometimes referred to as pellagra glove (affecting the dorsum of the hands) and pellagra boot (involving the feet and lower legs) dermatitis.

On examination, the rash had progressed to the dorsum of her feet, and she developed oromucosal ulceration. Around her neck, dermatitis appeared consistent with “Casal’s necklace” (Figures 1-2). She had not responded to prednisolone. Further history focusing on nutrition was taken, and it revealed that she had followed a very selective ketogenic regimen for several years on her own accord, hoping to improve her seizure symptoms. Based on this history, along with her symptoms and the characteristic “Casal’s necklace” rash, pellagra became the primary differential. The patient was also evaluated by the neurology and rheumatology teams. Neurological assessment revealed a mild sensory peripheral neuropathy, contributing to her unsteady gait. Their differential diagnosis included a nutritional deficiency or a paraneoplastic process, the latter of which was ruled out through further investigation. Rheumatological evaluation found that the case was not consistent with dermatomyositis; porphyria was also considered but subsequently excluded by a normal plasma porphyrin concentration.

The patient was started on nicotinamide 500 mg with intravenous infusion of a vitamin B collection and B12 injections. Prednisolone was slowly weaned off, and she was reviewed by the dietician. Within one month of treatment, her skin rash resolved completely. Over the following six months, she noticed significant improvements in peripheral neuropathy, dysphagia, and mental health. The clinical diagnosis of pellagra was confirmed by the patient’s substantial response to treatment.

## Discussion

Pellagra presents as dermatitis of sun-exposed areas of skin, beginning as an erythema with pruritus that may lead to vesiculation but more frequently becomes chronic, rough, scaly, and hard with crusts as a result of haemorrhage [2]. Our patient had the characteristic band of dermatitis encircling the neck, called "Casal's necklace," which is named after Don Gaspar Casal, whose description of pellagra was published in 1762 (Figure 1) [3]. The gastrointestinal tract may be involved with glossitis, stomatitis, gastroenteritis with diarrhoea, which is profuse, watery, and sometimes bloody [2]. Our patient had oromucosal ulceration and diarrhoea. Neurocognitive symptoms may present as anxiety, depression, tremor, and reduced or absent tendon reflexes; memory loss with pellagrous encephalopathy may occur in severe cases. Our patient had progressive generalised weakness and peripheral neuropathy. If the deficiency continues, pellagra can lead to death if left untreated for 4-5 years [4].

Pellagra is diagnosed primarily clinically in the appropriate context, with rapid response to supplementation supporting the diagnosis [1]. Primary pellagra is due to a nutritional deficiency of niacin or tryptophan. Secondary pellagra occurs from the failure of the body to utilise niacin/tryptophan available to it for effective metabolic functioning [5]. Our patient had a history of a prolonged restrictive diet, causing nutritional deficiency, and responded well to niacin supplementation and diversification of diet, supporting our diagnosis of primary pellagra. While primary pellagra has classically been associated with maize-based diets in endemic regions, our case illustrates a modern etiological shift toward voluntary dietary restrictions in developed nations. A similar case was reported by Ng and Neff, in which pellagra developed in a patient adhering to a restrictive, primarily vegan diet, further supporting this trend [6]. Notably, that patient primarily exhibited cutaneous manifestations and fatigue without the remaining classical triad of dermatitis, diarrhea, and dementia, as in our patient. A literature review of pellagra in patients with anorexia nervosa [7] and a retrospective observational study of pellagra patients with chronic alcoholism [8] demonstrated that although some patients present with the full clinical triad, it is not universally observed. This variability in presentation can contribute to delays in diagnosis, particularly in non-endemic settings where clinical suspicion may be low.

## Conclusions

Pellagra can often be misdiagnosed and can be fatal. This case highlights the importance of considering primary pellagra in patients who are at risk. Individuals who are homeless, have eating disorders, or follow restrictive diets can develop primary pellagra due to malnutrition, particularly in the context of various extreme diets gaining public popularity. There should be a low threshold for considering pellagra as a potential diagnosis early in patients with a photosensitive rash, cognitive decline, or gastrointestinal symptoms. Timely nutritional therapy can reverse symptoms and prevent a fatal outcome.
